# Preface

**DOI:** 10.2174/1570159X2301240904112839

**Published:** 2024-09-04

**Authors:** Fedinando Nicoletti

**Affiliations:** 1 Department of Pharmacology, University Sapienza of Rome, IRCCS Neuromed, Pozzilli, Italy

It is a great privilege to serve as the EIC of *Current Neuropharmacology* and to collaborate with Miss Rabia Moin and the team at Bentham Science Publishers on a daily basis. Miss Rabia Moin, in particular, has been of great help in coordinating all activities related to CN with great efficiency. In addition, Rabia is highly professional and polite, fully reflecting the style of the whole Bentham team. I am also grateful to all members of the editorial board, including the honorary advisor, co-editors, section editors, country editors, members of the editorial advisory board and the associate editorial board, executive guest editors, and guest editors, for their invaluable assistance in the screening and review process of manuscripts submitted to CN. They are all great scientists with established reputations in their fields of interest. I wish to express my gratitude to Prof. Akk (Washington University, School of Medicine, St. Louis, MO) for his kind and valuable help on several occasions.

For the coming year, we strongly encourage the submission of review articles, opinion articles, and position articles on drug treatment of neurological and psychiatric disorders. Proposals for Special Issues (Sis) are also strongly encouraged.

The following SIs have been published in 2024:

1. Is pain a symptom or a disease? How does the new evidence help to better understand this unsolved question?.
  by Dr. Massimo Allegri
2. Stress and stress-related disorders developmental and neuroendocrine aspects of stress and stress-related disorders (Part 1).
  by Dr. Agorastos Agorastos
3. Stress and stress-related disorders posttraumatic stress disorder (Part 2).
  by Dr. Agorastos Agorastos
4. Stress and stress-related disorders neurobiology & translational aspects of stress-related disorders (Part 3).
  by Dr. Agorastos Agorastos
5. Subcortical contribution to the role of the basal ganglia in action selection.
  by Dr. Veronique Coizet and Frederic Ambroggi

The following thematic issues are expected to be published in 2025:

1. Advanced progress in neuroimaging markers for neurological and psychiatric disorders.
  by Dr. Peipeng Liang
2. Addiction: A multi-faceted issue.
  by Dr. Fabrizio Schifano
3. The comorbidity of metabolic abnormality in psychiatric disorders: causes, risk factors, intervention, and healthcare trauma-related disorders: Neural circuitry, biomarkers, and therapeutic interventions.
  by Dr. Simone Battaglia
4. Translational informatics for neuropharmacology: database, ontologies and analytics.
  by Dr. Amjad Kamal
5. Biology redox: Homeostasis redox and redox cellular signalling in the brain.
  by Dr. Ramón Rama
6. Focus on ADHD: Navigating the complex landscape of ADHD and understanding the neurobiology, diagnosis, and interventions.
  by Dr. Beatrice Duthey
7. The role of psychoneuroimmune interaction in the pathogenesis of neuroinflammation.
  by Dr. Mikhail Melnikov
8. Therapeutic benefits of bioactive compounds in brain disorders.
  by Dr. Arianna Vignini
9. Developmental origins of health and disease (DOHaD).
  by Dr. Luciene Bruno Vieira
10. Novel insights on mechanisms and potential therapeutic targets for multiple sclerosis.
  by Dr. Smathorn Thakolwiboon
11. Mechanotransduction in brain vessel health and disease by concetta scimone. Mitoprotection and neurodegenerative diseases.
  by Dr. Sergey Bachurin
12. Microbiota-Gut-Brain axis in mood and neuropsychiatric disorders. Therapeutic opportunities.
  by Dr. István Bókkon
13. Intercellular communications in cerebral ischemia.
  by Dr. Zhong Chen
14. Emotion (Dys)Regulation: An integration of pharmacological, neurobiological and psychological frameworks.
  by Dr. Vladimir Kosonogov

We will do our best to further increase the quality of the journal and accelerate the review process and publication of accepted articles.

